# A simplified flow cytometric method for detection of inherited platelet disorders—A comparison to the gold standard light transmission aggregometry

**DOI:** 10.1371/journal.pone.0211130

**Published:** 2019-01-23

**Authors:** Kristoffer Navred, Myriam Martin, Lina Ekdahl, Eva Zetterberg, Nadine Gretenkort Andersson, Karin Strandberg, Eva Norstrom

**Affiliations:** 1 Coagulation Laboratory, Department of Clinical Chemistry, Division of Laboratory Medicine, Skåne County Council, Malmö, Sweden; 2 Department of Haematology, Coagulation Unit, Skåne University Hospital, Lund, Sweden; 3 Department of Translational Medicine, Lund University, Skåne County Council, Malmö, Sweden; University of Tsukuba, JAPAN

## Abstract

**Background:**

Flow cytometric platelet activation has emerged as an alternative diagnostic test for inherited platelet disorders. It is, however, labor intensive and few studies have directly compared the performance of flow cytometric platelet activation (PACT) to light transmission aggregometry (LTA). The aims of this study were 1/ to develop a simplified flow cytometric platelet activation assay using microtiter plates and 2/ to correlate the outcome to gold standard method LTA, and to clinical bleeding assessment tool scores (BAT score).

**Methods:**

The PACT method was developed in microtiter plates using adenosine diphosphate (ADP), collagen-derived peptide (CRP-XL) and thrombin receptor activator for peptide 6 (TRAP-6) as agonists. Antibodies against GPIIb-IIIa activation epitope (PAC1), P-selectin (CD62P) and lysosome-associated membrane glycoprotein 3 (LAMP3; CD63) were used as platelet activation markers. Sixty-six patients referred to the coagulation unit for bleeding symptoms were included in this single-center observational study. Platelet activation was determined by PACT and LTA. The results of both methods were correlated to BAT score.

**Results:**

A two-by-two analysis using Cohen’s kappa analysis gave moderate agreement between LTA and PACT (82%, kappa = 0.57), when PACT analysis with ADP and CRP-XL was compared to LTA. Using LTA as reference method, positive predictive value was 70% and negative predictive value was 87%. A substantial number of patients had high BAT score and normal LTA and PACT results. Patients with abnormal LTA or PACT results had higher BAT score than patients with normal results, but the difference was not significant.

**Conclusions:**

The performance in microtiter plates simplified the PACT method and enabled analysis of more patients at the same time. Our results indicate that with modification of the current PACT assay, a higher negative predictive value can be obtained. Furthermore, with comparable result to LTA the PACT could be used as a screening assay for inherited platelet disorders.

## Introduction

Inherited platelet function disorders (IPFD) is a heterogeneous group of disorders causing a propensity for bleeding. Its severity ranges from severe bleeding disorders such as Glanzmann’s thrombasthenia and Bernard Soulier’s syndrome to defects with only mild symptoms [1]. Defects can be found in the platelet glycoproteins, in receptors, in content or release of granules, in transcription factors and in signaling pathways [1]. Because of its complexity, the diagnostic work-up involves a wide range of methods and procedures, that are more or less accessible even to specialized clinical laboratories [2]. Recently the subcommittee of ISTH published a recommendation involving a step-wise approach to diagnosis of platelet disorders [3]. In the first step, facilitating a diagnosis in only 40% of IFPD patients, as much as 21–28 mL of whole blood is needed.

A key method in the diagnosis of platelet functional disorders is light transmission aggregometry (LTA) [4,5]. Light transmission is measured in platelet rich plasma (PRP) during agonist stimulation. Changes in light transmission correlate with the degree of platelet aggregation. By using a combination of different agonists, a wide range of platelet disorders can be investigated. Also, the release of ATP from the dense granules can be measured in the LTA by the use of chemilumniscence. LTA has the advantage that it is an established method and many of the known defects have been characterized using LTA [6,7]. It is therefore considered as the gold standard for diagnosis of many platelet dysfunctions [4]. A recommendation for the standardization of the LTA procedure has been published by the subcommittee of SSC/ISTH [5]. The major drawbacks of the method are the high amount of required blood making the method less optimal to do as screening in the pediatric population and its sensitivity to platelet count. LTA is not applicable for samples with a platelet count below 150 x 10^9^/L [5,8,9]. Thus, it is not feasible in patients with thrombocytopenia, which is often found in IFPD patients.

Flow cytometry has emerged as an alternative method for studying platelet activation [4,10–12]. It has an established role in the diagnostics of Bernard Soulier’s syndrome and Glanzman’s thrombasthenia, where the lack of receptors can easily be detected on the surface of resting platelets [13]. However, it is also used to study the activation of platelets by different agonists [10,14]. The exposure of specific antigens, such as the GPIIb-IIIa activation epitope (PAC1), P-selectin (CD62P) and LAMP-3 (CD63), on the platelet surface is measured after activation with a set of agonists. PAC1 is an antibody that specifically binds to the conformationally altered GPIIb-IIIa. The conformational changed occurs during platelet activation and is required for fibrinogen binding and platelet aggregation. CD62P is released from α-granules and CD63 from lysosomal and dense granules. The major advantage of flow cytometry is the small volume of blood sample required [12,15]. Also, it is less sensitive to platelet count and therefore even platelets from thrombocytopenic patients can be investigated [15]. However, it needs technically advanced instrumentation and the classical procedure involves many manual steps. Thus, it is time consuming, especially if the effect of different agonists and concentrations are investigated. Besides, its role in the diagnosis of IPFD is not as well established as LTA and therefore less has been published on the phenotypic patterns seen in different platelet disorders. Few comparisons to other more established methods have been published [16–18].

The aims of this study were 1/ to develop a simplified flow cytometric platelet activation assay using microtiter wells and 2/ to compare the outcome of this method to the outcome of LTA using a Born instrument in sixty-six consecutive patients admitted to a specialized coagulation unit for bleeding symptoms. Results of both PACT and LTA are also correlated to clinical bleeding assessment tool scores (BAT score, [19]).

## Materials and methods

Sixty-six consecutive patients referred to the coagulation unit were included in this retrospective study during a period of 20 months. Patients on medication with acetylsalicylic acid (ASA), P2Y12-inhibitors, non-steroidal anti-inflammatory (NSAID) products or selective serotonin reuptake inhibitors (SSRI) were excluded as well as patients with platelet count below 100 x 10^9^/L, since LTA is affected at lower platelet counts. Eighty-five percent were female and the median age was 33 years (range 8–78 years). BAT score was collected from the referral note, thus there were no need to access patient records. The study was approved by the Regional Ethical Review Board as a method comparison, Lund, Sweden (Dnr: 2015/886). Since the ethics committee considered the study as a methods comparison, they did not require an informed consent.

### Reagents

Vacutainer Citrate 4.5 mL tubes, Cellfix and antibodies PAC1-FITC, MsIgM-FITC and CD63-V450 BD were from BD Biosciences (Franklin Lakes, NJ, USA). Flow-Check, Flow-Set Fluorospheres and antibodies CD62P-PE, CD42b-APC and MsIgG-PE were from Beckman Coulter (Brea, CA, USA). Microtiter plates were from Bio-RAD (cat number CON960; CA, USA). Bovine serum albumin (BSA) was from Sigma-Aldrich (St Louis, MO, USA). ADP was from Sigma-Aldrich (St Louis, MO, USA), CRP-XL was purchased from Prof. Farndale (University of Cambridge, UK) and TRAP-6 was from Bachem (Bubendorf, Switzerland). Collagen and Epinephrine were from Chrono-Log Corp. (Haverton, PA, USA) and Ristocetin was from (American Biochemical & Pharmaceutical Corporation, Middlesex, U.K.). HEPES-bovine serum albumin (BSA) buffer (20 mM Hepes, 137 mM NaCl, 2.7 mM KCL, 1 mM MgCl and 1% BSA).

### Platelet activation assay using flow cytometry (PACT)

Blood samples were obtained from the antecubital vein using a vacutainer system. The first 4 mL were discarded and the blood sample was left on the bench for 20 minutes to avoid pre-activation of platelets. 20 μL of antibody cocktail containing PAC1-FITC, CD62P-PE and CD63-V450 were aliquoted in microtiter wells. The final volume of antibody in each well was 5 μL PAC1-FITC, 5 μL CD62P-PE and 2.5 μL CD63-V450 that were diluted in HEPES-bovine serum albumin (BSA) buffer. In the first well control antibodies (MsIgM-FITC and MsIgG-PE) were added instead of activation markers. 20 μL of citrated blood was incubated for 15 minutes in the dark with 20 μL of the platelet gating marker CD42b-APC in 200 μL HEPES-BSA buffer. During incubation, agonist dilutions (ADP, final concentrations 5 and 0.25 μM, CRP-XL final concentrations 20 μg/mL and 2 μg/mL and TRAP-6 final concentrations 25 μM and 5 μM) were prepared in 1.2 mL tubes and 40 μL of the dilutions were added to the microtiter wells. The blood labelled with CD42b-APC was further diluted in 600 μL HEPES-BSA and aliquoted into eight 1.2 mL tubes. To start the assay, 40 μL of CD42b-APC labelled blood was added to each microtiter well using multi-pipets. The mixture was incubated at room temperature in dark for 15 minutes before Cellfix (final concentration 0.3%) was added to stop the reaction and to avoid dissociation of the PAC1-antibody.

The samples were diluted in 500 μL Isoflow and run on flow cytometer, Gallios (Beckman Coulter, Brea, CA, USA) and Navios (Beckman Coulter, Brea, CA, USA). The laser setting W^2^ was used, logarithm scale was used for forward scatter, side scatter and all fluorescence detectors. Threshold for forward scatter and side scatter were set to two with live gate on CD42b-APC. Flow-Check and Flow-Set Fluorospheres were used for daily quality control to ensure stability of instrument fluidics and optical alignment as well as standardization of fluorescence intensity and light scatter. The flow cytometry data were analyzed using Kaluza Flow Cytometry Software (Beckman Coulter, Brea, CA, USA). Activation was expressed as percentage of positive platelets of the whole platelet population. Platelets are easily activated during sample preparation and early expose CD62P, i.e. resting platelets are not optimal to define the true negative events for this marker. Instead the MsIg-PE isotype control was applied and set to 2% for CD62P-PE. PAC1 can also become exposed on pre-activated platelets, however, to a much lesser extent. Since the fluorescent density of the MsIgM-FITC isotype control differed from that of PAC1-FITC, resting platelets were set to 2% to define negative events. CD63 becomes only exposed on strongly activated platelets and thus resting platelets are optimal to define the negative population for CD63-V450 and were thus set to 2%. To establish normal reference values, blood from 20 healthy individuals with no prior history of bleeding symptoms, were run. The age of normal persons ranged from 22 to 65 years of age and there were 50% men and 50% women. The normal subjects had not taken acetylsalicylic acid (ASA), P2Y12-inhibitors, non-steroidal anti-inflammatory (NSAID) products or selective serotonin reuptake inhibitors (SSRI) for the last 10 days. The cut-off limit was set to -2SD. Samples from healthy individuals were thereafter run with a frequency of 1–4 weeks as controls and were all within the normal range.

### Light transmission aggregometry (LTA)

LTA was performed on a Chrono-Log model 700 lumi aggregometer (Chrono-Log corp.). For LTA four tubes of citrated blood were drawn from each subject. PRP was obtained by centrifugation at 200 g for 10 minutes in room temperature. PRP was pipetted into a new tube using plastic pipet. In order to avoid the transfer of other cells than platelets, the lowest part of the plasma (approximately 1 cm) was left in the citrated tube. Platelet poor plasma was obtained by an additional centrifugation of the citrated tube at 2500 g for 10 minutes. LTA was performed according to the ISTH SSC guidelines (5). Final concentrations were ADP 2 μM, Collagen 2 μg/mL, Epinephrine 5 μM and Ristocetin 1.2 mg/mL.

### Statistical analysis

Statistical analysis was performed using GraphPad Prism 7.0d. Degree of consistency between LTA and PACT was calculated using Cohen’s kappa test. Mann-Whitney U test was performed to compare the median BAT score of normal versus abnormal results in LTA or PACT. To demonstrate the diagnostic performance of different parameters in PACT versus LTA, ROC analysis was performed.

## Results

### Establishment of a microtiter assay

**Fig 1:**
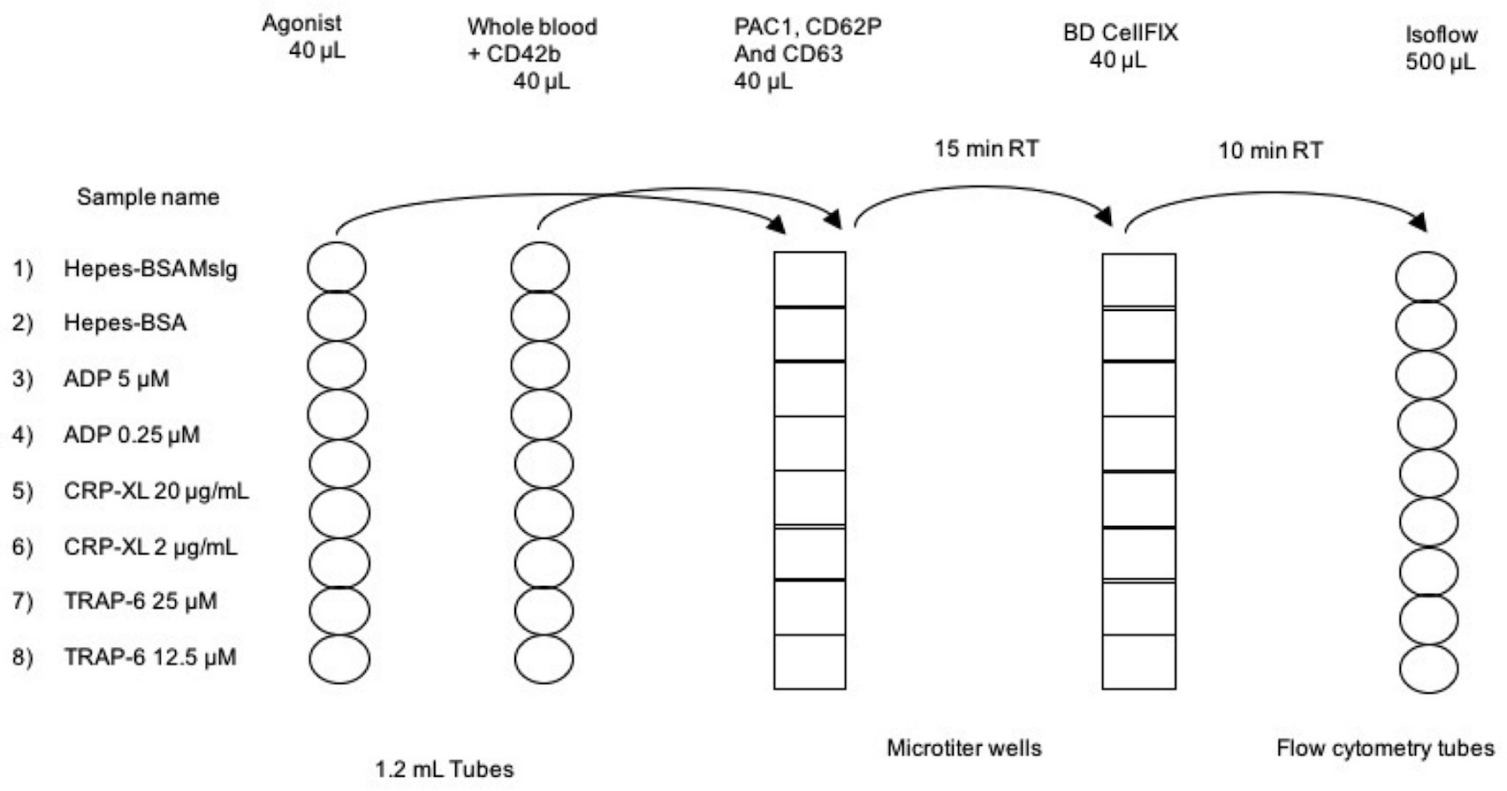
The experimental set-up for the PACT assay. The activation markers PAC1, CD62P and CD63 were aliquoted into microtiter wells. Agonist dilutions and whole blood pre-incubated with CD42b was added to the microtiter wells to start the assay. After 15 minutes incubation, the assay was stopped by transferring the mix to wells containing BD Cellfix. After additional 10 minutes, the fixated samples were added to IsoFlow before flow cytometric analysis. All transfers are done with multipipets.

**Fig 2:**
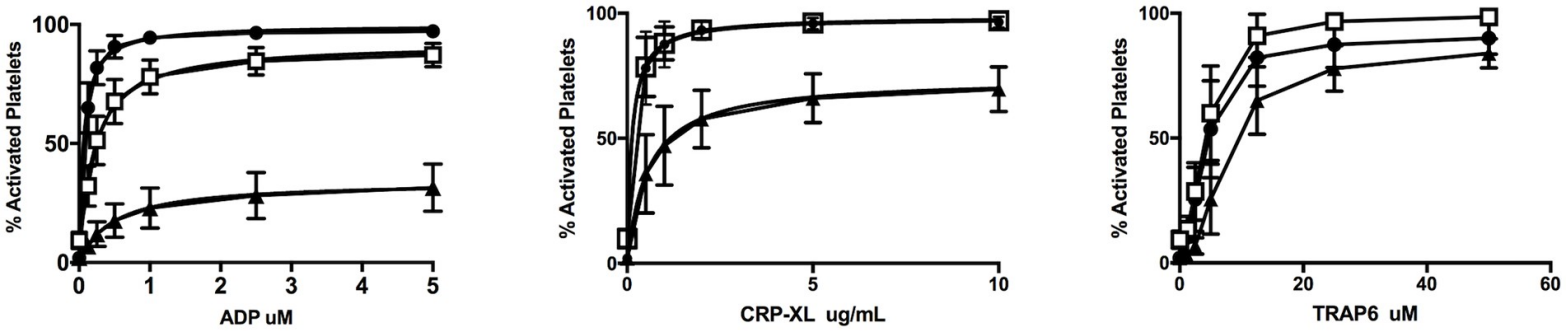
Platelet activation in healthy subjects. Platelet activation was performed on platelets from 20 healthy individuals using ADP (0–5 μM), CRP-XL (0–20 μg/ml) and TRAP-6 (0–50 μM). The expression of PAC1 (closed circles), CD62P (open squares) and CD63 (closed triangles) are shown as percentage of activated platelets and the graph shown are mean calculated from results from the 20 healthy individuals.

Fig 1 shows the experimental set-up of the platelet activation assay. The use of fixation or the choice of tubes may affect the amount of pre-activated platelets. However, when we compared the CD62P exposure on resting platelets from 10 different healthy individuals there were no differences; 10% activated in microtiter wells with fixation (SD = 3.7), compared to 14% in tubes without fixation (SD = 5). However, in the non-fixed platelets we saw a decrease in the PAC1 signal, if the samples were not analyzed immediately. Fig 2 shows the titration of ADP, CRP-XL and TRAP-6 in PACT assay using samples from 20 healthy individuals.

### Intra-individual variation of PACT assay

Samples from one healthy individual were analyzed in the PACT assay three times during a period of six months to enable calculation of intra-individual CV of the method (Table 1). The variation of CD63 expression after ADP stimulation was not acceptable (40%), and also at the lower concentration of ADP (0.25 μM) the CV was high (22%) for expression of P-selectin. For the other combinations of activation markers and agonists, the CVs were below 15% and a tendency was seen for all agonists that the lower concentration gave the highest variation. Clinical analysis of samples was limited to those combinations of agonist and activation markers with CV beneath 15%.

**Table 1:** Intra-individual variation of PACT assay. (n = 3 replicates).

	ADP		CRP-XL		TRAP-6	
	5 μM	0.25 μM	20 μg/mL	2 μg/mL	25 μM	5 μM
**PAC1**	1.1	8.7	0.4	0.4	2.6	4.6
**CD62P**	8.4	22	0.5	1.9	0.2	5.2
**CD63**	40	40	6.2	12	1.8	15

### Use of two concentrations of agonists

To investigate the benefit of using two concentrations of each agonist, we compared the outcome of the different concentrations. Because of the high variation (CV%) of CD63 expression after ADP stimulation and P-selectin expression at 0.25 μM, expression of PAC1 was solely investigated after ADP stimulation. Only in one case the low concentration of agonist was abnormal, whereas the high concentration of agonist gave normal results (PAC1 expression after TRAP-6 stimulation). In all other cases with aberrant results, the high concentration was abnormal or both high and low concentrations were abnormal.

### LTA versus PACT

Thirty percent of the patients (20 of 66) had an abnormal response in LTA. All cases with abnormal LTA results showed a poor response to ADP, either as the only aberrant result or in combination with low response to collagen or epinephrine or a poor ATP release. The most frequent result was a reversible ADP wave (11 of 20 cases). Forty-four percent of the patients (29 of 66) had an abnormal result in PACT. The most frequent finding was a low response to TRAP-6 (9 of 29), ADP (7 of 29) or a combination of ADP and TRAP-6 (4 of 29).

We compared PACT results using agonists ADP and CRP-XL with the outcome of LTA (Table 2). Using Cohen’s kappa test there was an 82% agreement between LTA and PACT (kappa = 0.57). Using LTA as reference method, the positive predictive value was 70% whereas the negative predictive value was higher, 87%.

**Table 2:** Agreement between light-transmission aggregrometry and the results of ADP and CRP-XL stimulation in flow cytometry. “Abnormal” and “normal” LTA indicates the presence respectively absence of abnormal aggregation wave and/or ATP release, “abnormal” and “normal” flow cytometry indicates abnormal respectively normal response in flow cytometry using -2 SD as cut-off limit. A two-by-two analysis of the agreement using Cohen’s kappa analysis gives an 82% agreement with a kappa of 0.57.

	LTA abnormal	LTA normal	
**Flow cytometry abnormal**	14	6	PPV = 70%
**Flow cytometry normal**	6	40	NPV = 87%
	Sensitivity = 70%	Specificity = 87%	

The results of PACT-TRAP-6 were also analyzed against LTA (Table 3). Eight of nine cases with abnormal TRAP-6 as the only flow cytometric finding were normal in LTA. Nine of the twenty patients with a decreased TRAP-6 response, either in combination with other agonists, or as the only finding, were LTA abnormal. Among the patients with abnormal LTA and a defect response to more than one agonist in PACT, two were confirmed to have Glanzman’s thrombastenia (by flow cytometry analysis of CD41, the patients were siblings), four cases were also abnormal in ADP response. In two cases the results indicated a release defect, since CD63 and/or CD62P was low with more than one agonist. In the group of LTA normal and TRAP-6 abnormal in combination with other agonists, the results in two cases indicated a release defect and one had a combination of low ADP and TRAP-6 response.

**Table 3:** Results of TRAP-6 analysis with flow cytometry as compared to the LTA results.

	TRAP-6 abnormal	TRAP-6 + ADP and/or CRP-XL abnormal
**LTA normal**	8	3
**LTA abnormal**	1	8

**Fig 3:**
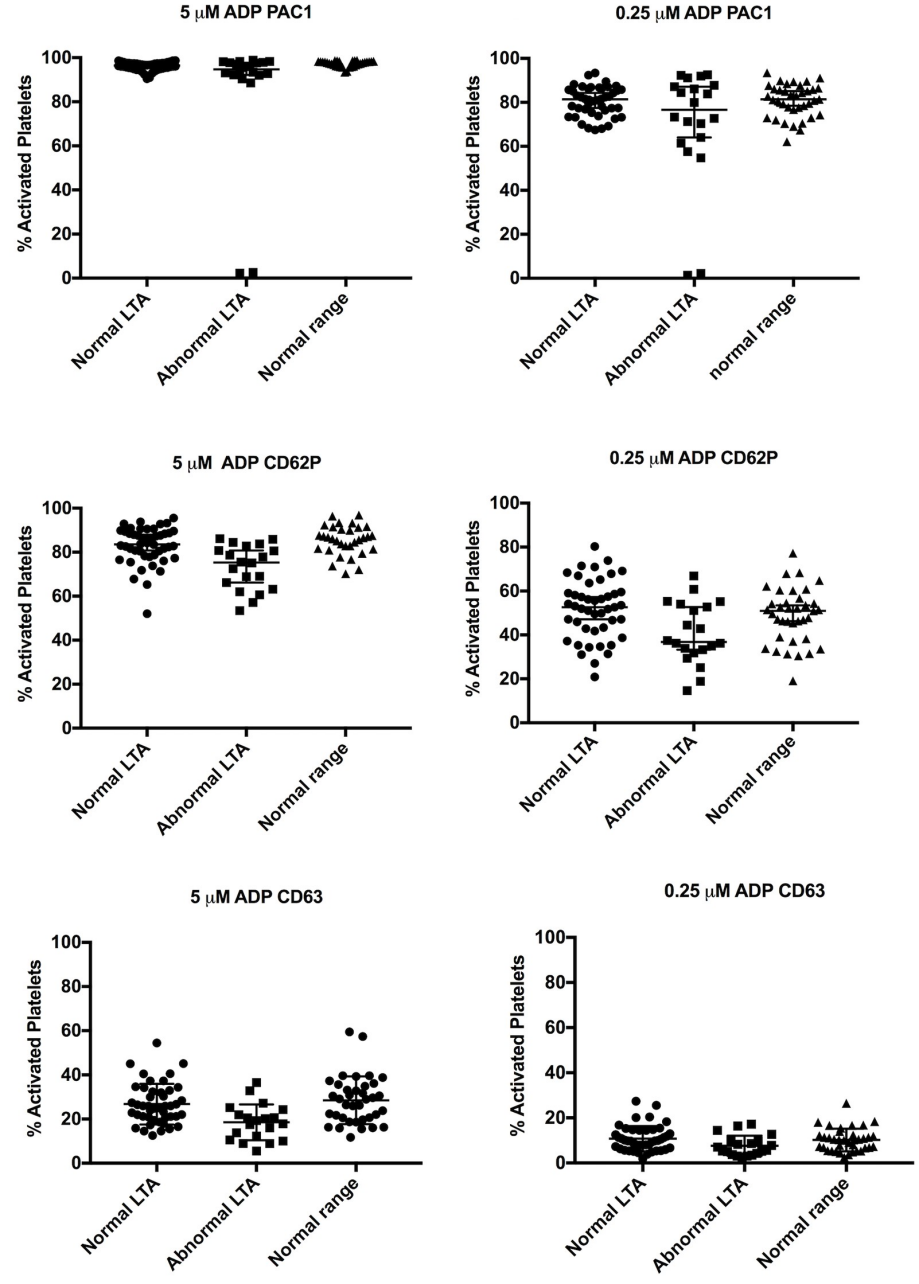
Comparison of PACT-ADP to LTA. The expression of activation markers after ADP stimulation in flow cytometry compared to LTA. Error bars show median value with SD. Mann-Whitney U test was performed, median values and p-values are listed in Table 4.

**Fig 4:**
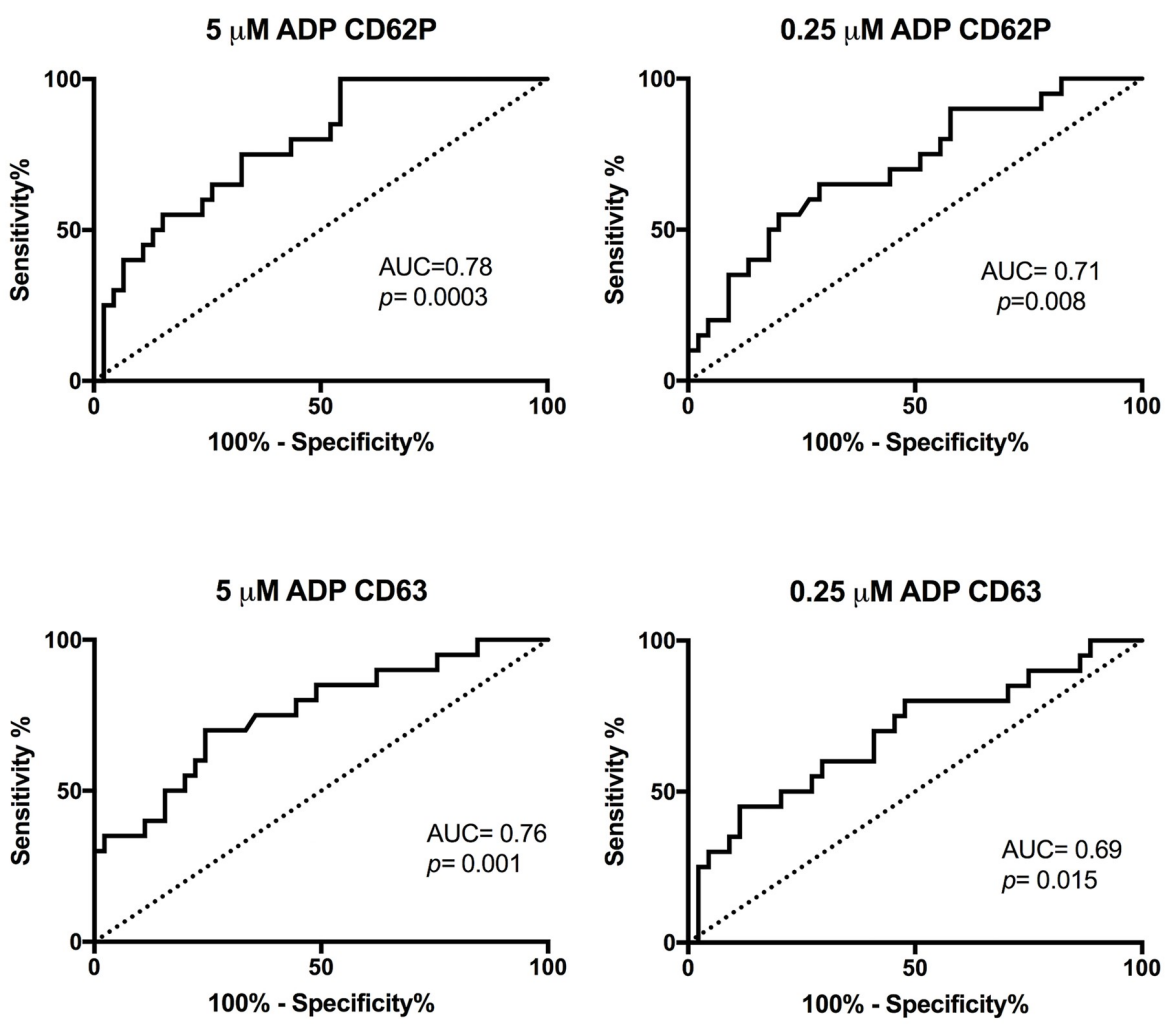
ROC analysis of ADP-CD62P and ADP-CD63 as compared to LTA. ROC analysis was performed after ADP stimulation for activation markers CD62P and CD63 as compared to ADP response in LTA. Area under curve (AUC) and *p*-values are listed in the figures.

Since all of the abnormal LTA showed low response to ADP, activation markers after ADP stimulation was compared to negative or positive LTA results (Fig 3). Median values for expression of P-selectin at both ADP concentrations were significantly lower for the cases with abnormal LTA results than the cases with normal results (Table 4). The median value for expression of CD63 was also significant lower for the cases with abnormal LTA results compared to normal LTA results (Table 4). The median values for PAC1 expression was not significantly different between abnormal and normal LTA cases (Table 4). ROC analysis was performed for the activation markers P-selectin and CD63 (Fig 4). P-selectin at 5 μM ADP showed the best agreement with the LTA results (AUC = 0.78).

**Table 4:** Summary of comparison PACT-ADP and LTA.

	ADP 5 uM			ADP 0.25 uM		
	LTA normal (Median)	LTA abnormal (Median)	*p-value*	LTA normal (Median)	LTA abnormal (Median)	*p-value*
**PAC1**	97	95	0.23	81	77	0.39
**CD62P**	84	75	0.0002	53	37	0.007
**CD63**	26	19	0.0007	9.8	6.4	0.015

Mann-Whitney U test: Expression of PAC1, CD62P (P-selectin) and CD63 (LAMP3) after activation with ADP (5 μM or 0.25 μM) expressed as % activated platelets are compared to the results of LTA.

**Fig 5:**
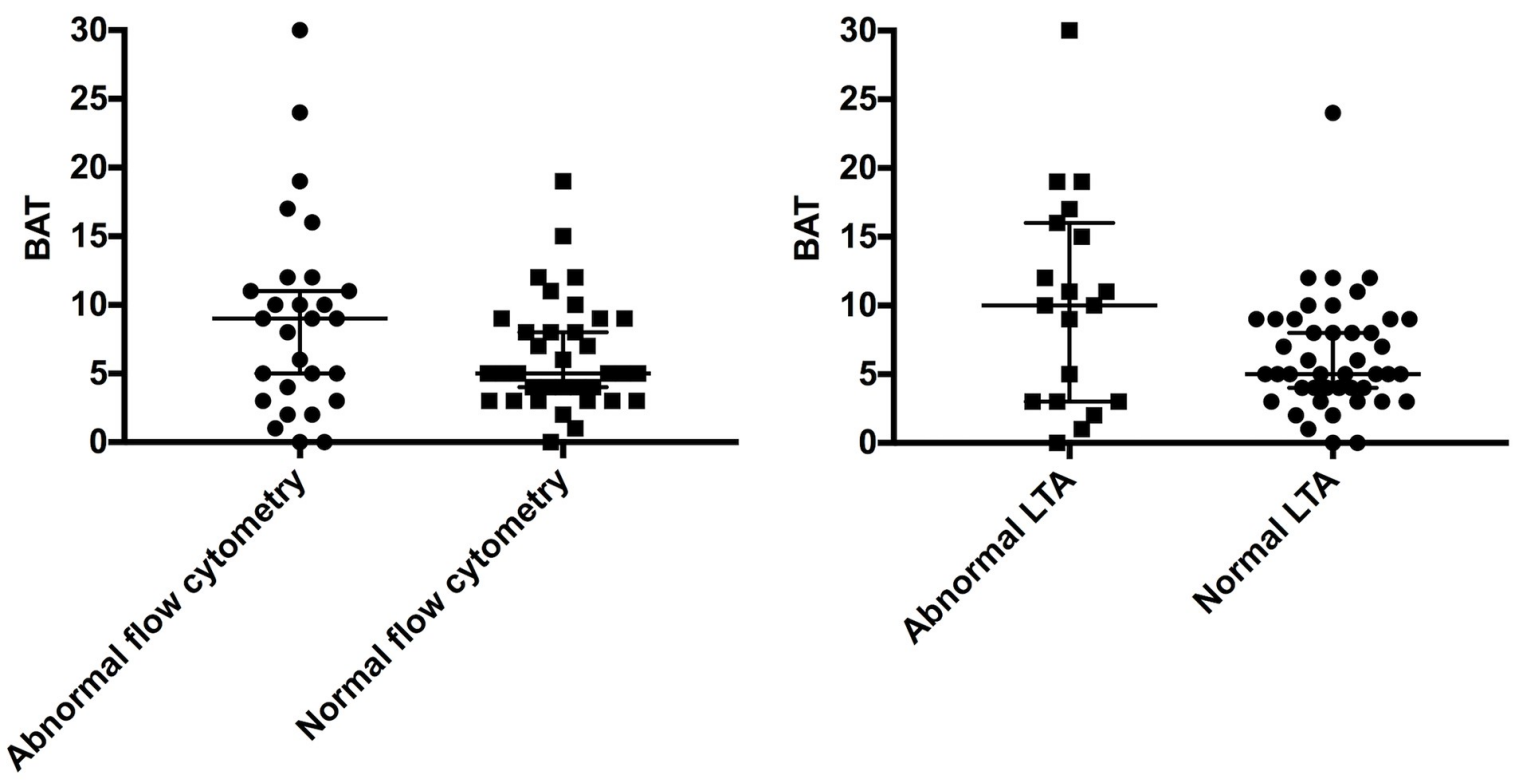
Comparison between BAT score and flow cytometry or LTA. The results were grouped in abnormal or normal flow cytometry (left panel) and LTA (right panel) and plotted against the clinical assessment bleeding tool score (BAT). Abnormal versus normal flow cytometry: *p-value* 0.14. Abnormal versus normal LTA: *p-value* 0.07.

The association to bleeding score was also compared for both LTA and PACT (Fig 5). For both flow cytometry and LTA, a large number of normal cases had high BAT score. However, the median value of BAT for abnormal results was higher than for the normal results for both methods, but with no significant difference. ROC analysis was performed for both LTA and PACT as compared to BAT and showed low accuracy for both LTA and PACT (AUC = 0.64, *p* = 0.07 for LTA and AUC = 0.61, *p* = 0.14 for PACT).

## Discussion

The study of platelet dysfunction is complex, because of the heterogeneity of the underlying pathophysiology. Therefore, no single method has been identified as a simple screening diagnostic for platelet dysfunctions. The flow cytometric method has the advantages that it only requires small amounts of blood and that it can be analyzed even in thrombocytopenic patients [14]. However, it is time-consuming, requires many manual steps and lacks standardization. The use of microtiter plates simplified the method and allowed higher throughput, the possibility to run more samples and more concentrations of agonists at the same time.

One of the weakness of PACT assays is the lack of published experience in a clinical setting. There are only a few studies that have compared the method to other traditional aggregation methods [16,17]. Dovlatova et al. compared traditional LTA with a whole blood remote flow cytometric-based platelet function test in 61 study patients and found an 84% concordance between the two methods (kappa = 0.668). The association to bleeding score was not reported. Asten et al. compared the performance of PACT assay to LTA using PAP8E aggregometer and found fair agreement between LTA and PACT. Their results also showed an association to bleeding score. We observed a moderate agreement (kappa = 0.57) between LTA and PACT with a concordant result in 82% of the cases.

Consistent with the results of Asten et al. we observed an association to BAT score for both PACT analysis and LTA, although not significant. However, since generally patients with high bleeding score were selected, the association to bleeding score should be interpreted with caution. Consistent with results from other studies, the number of patients with high bleeding score and normal LTA and PACT score is high, indicating either that the methods do not detect all platelet dysfunctions or that BAT score, developed for VWD detection, has shortcomings in this patient group. In a survey performed by the SSC of ISTH, it was revealed that at the best 40% of patients with suspected platelet dysfunction were identified using existing methods in the first line of investigation [2]. This also reflects the heterogeneity of the disease and the difficulty to mimic the physiological function of platelets in a laboratory set up.

The methodology of LTA and flow cytometry are in many aspects different. Aggregation is run on undiluted platelet rich plasma, whereas flow cytometry is run on blood diluted 40 times. The measured end-points are on the one hand small molecular changes to receptors and antigens and on the other hand aggregation of the platelets. The dynamics in the two methods are dissimilar and it is likely that they may show different sensitivity dependent on the defect in question. The use of collagen in LTA versus CRP-XL in flow cytometry is also not completely comparable, since CRP-XL is specific for the GPVI receptor [20]. CRP-XL is however the preferred agonist in flow cytometry, due to the risk of pseudo-aggregates when using collagen. Likewise, CD63 is a marker of both, dense granules and lysosomal granules and does not measure the content of the granules. Therefore, it is insensitive to empty sack syndrome, where the dense granules exist, but do not contain ADP/ATP [21]. Since LTA measures overall platelet aggregation, this method is very sensitive to NSAID, whereas flow cytometry is not significantly affected when ADP, CRP-XL and TRAP-6 are used as agonists in such patients.

Interestingly, we found a high negative predictive value (87%) when PACT was compared to LTA. Since PACT only requires 20 μL as compared to 4 citrated 4.5 mL whole blood tubes in LTA, a high negative predictive value can implicate a potential to use PACT as a screening assay before LTA. This would be especially beneficial in the pediatric patients. Therefore, modifications of the PACT assay to increase the NPV even further would be ideal. Since the use of two concentrations of agonists did not give any more value than 1 concentration, one concentration of each agonists could be substituted with other agonists, widening the range of possible detected defects. In particular, our experience has shown that patients on medication with ASA most often show normal results in our PACT method but positive results in LTA. Thus, the panel should include an AA analog to cover inherited defects in the arachidonic acid pathway or exclude non-reported use of ASA. This would also be in accordance to the first line of agonist recommended from SSC. Epinephrine would also be preferable to include, since it is also used in LTA for evaluation of a second wave. Our results with TRAP-6 in PACT indicate that a broader range of agonists may increase the possibility to detect defects. In most cases of only TRAP-6 positive results, the LTA showed negative results, which could be expected, since TRAP-6 is not routinely tested in LTA.

In conclusion, we report a simplified PACT assay with higher throughput and with moderate agreement with the gold standard LTA. Further modification to increase the negative predictive value as compared to LTA would open the possibility to use PACT as a single screening assay before LTA. By including more agonists that usually are analyzed in LTA such as arachidonic acid and epinephrine, this can be accomplished. Especially in the pediatric patients this is a significant improvement.
